# Quaternary tryptammonium salts: *N*,*N*-dimethyl-*N*-*n*-propyl­tryptammonium (DMPT) iodide and *N*-allyl-*N*,*N*-di­methyl­tryptammonium (DMALT) iodide

**DOI:** 10.1107/S2056989020010014

**Published:** 2020-07-24

**Authors:** Andrew R. Chadeayne, Duyen N. K. Pham, James A. Golen, David R. Manke

**Affiliations:** a CaaMTech, LLC, 58 East Sunset Way, Suite 209, Issaquah, WA 98027, USA; b University of Massachusetts Dartmouth, 285 Old Westport Road, North Dartmouth, MA 02747, USA

**Keywords:** crystal structure, tryptamines, indoles, hydrogen bonding

## Abstract

The solid-state structures of two quaternary trytpammonium salts, namely, *N*,*N*-dimethyl-*N*-*n*-propyl­tryptammonium (DMPT) iodide [systematic name: 2-(1*H*-indol-3-yl)-*N*,*N*-dimethyl-*N*-propyl­aza­nium iodide], C_15_H_23_N_2_^+^·I^−^, and *N*-allyl-*N*,*N*-di­methyl­tryptammonium (DMALT) iodide, [systematic name: 2-(1*H*-indol-3-yl)-*N*,*N*-dimethyl-*N*-(prop-2-en-1-yl)aza­nium iodide], C_15_H_21_N_2_^+^·I^−^, are reported. Both salts possess a tri­alkyl­tryptammonium cation and an iodide anion in the asymmetric unit, which are joined together through N—H⋯I inter­actions. The DMALT structure was refined as an inversion twin, and the allyl group is disordered over two orientations with a 0.70 (4):0.30 (4) ratio.

## Abstract 2

The solid-state structures of two quaternary tryptammonium salts, *N*,*N*-dimethyl-*N*-*n*-propyl­tryptammonium (DMPT) iodide and *N*-allyl-*N*,*N*-di­methyl­tryptammonium (DMALT) iodide are reported.

## Chemical context   

Quaternary tryptammonium salts have been observed in nature going back to 1934 when bufotenidine, the *N*-trimethyl analogue of serotonin, was discovered in the excretions of toads (Wieland *et al.*, 1934 ▸). The unsubstituted *N*,*N*,*N*-tri­methyl­tryptammonium iodide was studied in 1936 and demonstrated nicotine-stimulating action (Lee *et al.*, 1936 ▸). In 1987, Gartz first identified a quaternary tryptammonium in ‘magic mushrooms’ when he isolated aeruginascin, *N*,*N*,*N*-trimethyl-4-phospho­ryloxytryptamine (Gartz, 1987 ▸). The tryptamines of ‘magic mushrooms’ have garnered a great deal of inter­est of late as their psychotropic activity is being explored for the treatment of mental disorders including depression and anxiety (Johnson & Griffiths, 2017 ▸; Daniel & Haberman, 2017 ▸). Aeruginascin, in particular, has been featured in popular media for its potential to modulate the activity of psilocybin through an entourage effect (Farah, 2018 ▸), as well as its possible involvement in wood-lovers paralysis (Revell, 2020 ▸). The recent synthesis of aeruginascin (Sherwood, *et al.* 2020 ▸) and its active metabolite, 4-hy­droxy-*N*,*N*,*N*-tri­methyl­tryptamine (Chadeayne, Pham, Reid *et al.*, 2020 ▸), as well as the biosynthetic production of both (Milne *et al.*, 2020 ▸) further demonstrate the attention that these mol­ecules have received. To this end, we sought to explore new quaternary tryptammonium salts, and the syntheses and structures of *N*,*N*-dimethyl-*N*-*n*-propyl­tryptammonium (DMPT) iodide and *N*-allyl-*N*,*N*-di­methyl­tryptammonium (DMALT) iodide are reported.
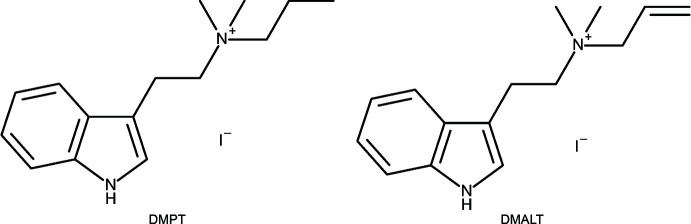


## Structural commentary   

**Figure 1:**
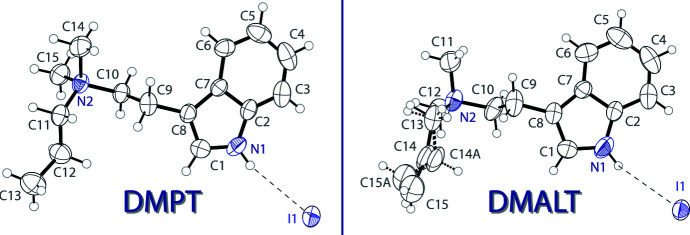
The mol­ecular structure of DMPT iodide (left) and DMALT iodide (right), showing the atomic labelling. Displacement ellipsoids are drawn at the 50% probability level. Dashed bonds indicate a disordered component in the structure. Hydrogen bonds are shown as dashed lines.

The mol­ecular structure of DMPT iodide is shown on the left of Fig. 1 ▸. The asymmetric unit contains one *N*,*N*-dimethyl-*N*-*n*-propyl­tryptammonium (C_15_H_23_N_2_^+^) cation and one iodide anion. The indole ring of the cation is near planar, with a mean deviation from planarity of 0.011 Å. The ethyl­ammonium arm is turned away from the plane with a C7—C8—C9—C10 torsion angle of 89.1 (4)°. The mol­ecular structure of DMALT iodide is shown on the right of Fig. 1 ▸. The asymmetric unit contains one *N*-allyl-*N*,*N*-di­methyl­tryptammonium (C_15_H_21_N_2_^+^) cation and one iodide anion. The indole ring of the cation is near planar, with a mean deviation from planarity of 0.013 Å. The ethyl­ammonium arm is turned away from the plane with a C7—C8—C9—C10 torsion angle of 101.8 (10)°. The allyl group is disordered over two orientations with a 0.30 (4) to 0.70 (4) occupancy ratio for C14, C15 and C14*A*, C15*A*, respectively.

## Supra­molecular features   

**Figure 2:**
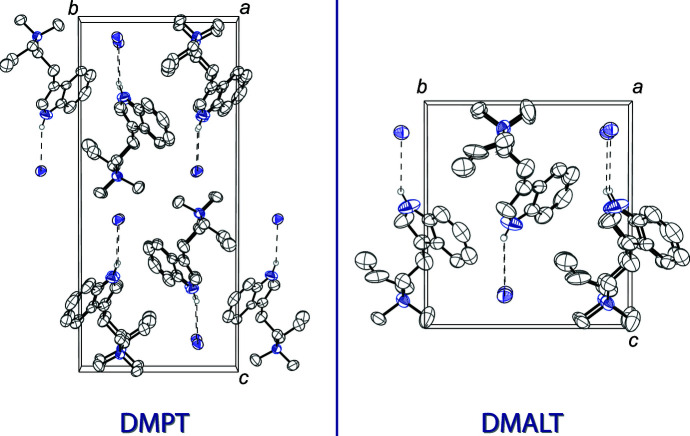
The crystal packing of DMPT iodide (left), viewed along the *a* axis, and the crystal packing of DMALT iodide (right), viewed along the *a* axis. The hydrogen bonds (Tables 1 ▸ and 2 ▸) are shown as dashed lines. Displacement ellipsoids are drawn at the 50% probability level. Hydrogen atoms not involved in hydrogen bonds are omitted for clarity. Only one component of the allyl disorder is shown in the DMALT structure.

The DMPT cation and the iodide anion are held together in the asymmetric unit *via* N1—H1⋯I1 hydrogen bonds, between the indole nitro­gen and the iodide (Table 1 ▸). The packing of DMPT iodide is shown at the left of Fig. 2 ▸. The DMALT structure is very similar to that of DMPT, possessing a very similar unit cell with half of the volume. The cation and anion are held together in the asymmetric unit through N1—H1⋯I1 hydrogen bonds (Table 2 ▸). The packing of DMALT iodide is shown on the right of Fig. 2 ▸

## Database survey   

Only two other quaternary tryptammonium structures have been reported, and are those of 4-hy­droxy-*N*,*N*,*N*-tri­methyl­tryptammonium (4-HO-TMT) iodide and 4-acet­oxy-*N*,*N*,*N*-tri­methyl­tryptammonium (4-AcO-TMT) iodide, whose structures demonstrate different packing including the oxygen atoms of the compounds (XUXFAA and XUXDUS: Chadeayne, Pham, Reid *et al.*, 2020 ▸). The other most closely related structures reported are of the *N*,*N*,*N*-trimethyl deriv­ative of tryptophan – hypaphorine. This includes the zwitterionic hypaphorine (IZUTUU: Arderne & Ndinteh, 2016 ▸), its hydro­iodide salt (PAMRUQ: Jones & Tiekink, 1997 ▸), and its 6-bromo derivative (BHYPUR: Raverty *et al.*, 1977 ▸). DMPT iodide is synthesized from the freebase of *N*-methyl-*N*-propyl­tryptamine (MPT), whose structure has been reported (WOHYAW: Chadeayne *et al.*, 2019 ▸). DMALT iodide is synthesized from *N*-allyl-*N*-methyl­tryptamine (MALT), whose structure has been reported as its fumarate salt (GUPBOL; Chadeayne, Pham, Golen & Manke, 2020 ▸).

## Synthesis and crystallization   

*N*,*N*-dimethyl-*N*-propyl­tryptammonium iodide was prepared by mixing 101 mg of a commercial sample of *N*-methyl-*N*-propyl­tryptamine (The Indole Shop) and 4 mL of methyl iodide in 4 mL of methanol. The mixture was refluxed for twelve hours under an atmosphere of nitro­gen. The solvent was removed *in vacuo*, and the remaining residue was recrystallized from ethanol to yield colourless single crystals suitable for X-ray diffraction studies. The product was also characterized by nuclear magnetic resonance. ^1^H NMR (400 MHz, D_2_O): δ 7.69 (*d*, *J* = 8.0 Hz, 1 H, Ar*H*), 7.55 (*d*, *J* = 8.2 Hz, 1 H, Ar*H*), 7.33–7.28 (*m*, 2 H, Ar*H*), 7.22 (*t*, *J* = 7.0 Hz, 1 H, Ar*H*), 3.60 (*m*, 2 H, C*H*_2_), 3.36 (*m*, 4 H, C*H*_2_), 3.17 (s, 6 H, C*H*_3_), 1.82 (m, 2 H, C*H*_2_), 0.97 (*t*, *J* = 7.0 Hz, 3 H, C*H*_3_).

*N*-allyl-*N*,*N*-di­methyl­tryptammonium iodide was prepared by mixing 101 mg of a commercial sample of *N*-allyl-*N*-methyl­tryptamine (The Indole Shop) with 4 mL of methyl iodide in 4 mL of methanol. The mixture was refluxed for twelve hours under an atmosphere of nitro­gen. The solvent was removed *in vacuo*, and the remaining residue was recrystallized from acetone to yield coloruless crystals suitable for X-ray diffraction studies. The product was also characterized by nuclear magnetic resonance. ^1^H NMR (400 MHz, D_2_O): δ 7.69 (*d*, *J* = 7.8 Hz, 1 H, Ar*H*), 7.55 (*d*, *J* = 8.2 Hz, 1 H, Ar*H*), 7.32–7.28 (*m*, 2 H, Ar*H*), 7.22 (*t*, *J* = 7.2 Hz, 1 H, Ar*H*), 6.13–6.03 (*m*, 1 H, C*H*), 5.77–5.71 (*m*, 2 H, C*H*_2_), 4.04 (*d*, *J* = 7.3 Hz, 2 H, C*H*_2_), 3.61–3.56 (*m*, 2 H, C*H*_2_), 3.37–3.32 (*m*, 2 H, C*H*_2_), 3.17 (*s*, 6 H, C*H*_3_).

## Refinement   

Crystal data, data collection and structure refinement details are summarized in Table 3 ▸.

## Supplementary Material

Crystal structure: contains datablock(s) DMPT, DMALT. DOI: 10.1107/S2056989020010014/zq2255sup1.cif

Structure factors: contains datablock(s) DMPT. DOI: 10.1107/S2056989020010014/zq2255DMPTsup2.hkl

Structure factors: contains datablock(s) DMALT. DOI: 10.1107/S2056989020010014/zq2255DMALTsup3.hkl

Supporting information file. DOI: 10.1107/S2056989020010014/zq2255DMPTsup4.cml

Supporting information file. DOI: 10.1107/S2056989020010014/zq2255DMALTsup5.cml

CCDC references: 2017818, 2017817

Additional supporting information:  crystallographic information; 3D view; checkCIF report

## Figures and Tables

**Table 1:** Hydrogen-bond geometry (Å, °) for DMPT[Chem scheme1]

*D*—H⋯*A*	*D*—H	H⋯*A*	*D*⋯*A*	*D*—H⋯*A*
N1—H1⋯I1	0.86 (1)	2.91 (2)	3.733 (3)	162 (3)

**Table 2:** Hydrogen-bond geometry (Å, °) for DMALT[Chem scheme1]

*D*—H⋯*A*	*D*—H	H⋯*A*	*D*⋯*A*	*D*—H⋯*A*
N1—H1⋯I1	0.86	2.95	3.727 (6)	152

**Table 3:** Experimental details

	DMPT	DMALT
Crystal data		
Chemical formula	C_15_H_23_N_2_^+^·I^−^	0.5C_15_H_21_N_2_^+^·0.5I^−^
*M*_r_	358.25	178.12
Crystal system, space group	Monoclinic, *P*2_1_/*c*	Monoclinic, *P*2_1_
Temperature (K)	303	303
*a*, *b*, *c* (Å)	7.4471 (6), 9.9016 (9), 22.052 (2)	7.3471 (8), 9.9672 (9), 10.9499 (11)
β (°)	94.184 (3)	94.671 (3)
*V* (Å^3^)	1621.8 (2)	799.20 (14)
*Z*	4	4
Radiation type	Mo *K*α	Mo *K*α
μ (mm^−1^)	1.96	1.99
Crystal size (mm)	0.40 × 0.14 × 0.12	0.39 × 0.22 × 0.15

Data collection		
Diffractometer	Bruker D8 Venture CMOS	Bruker D8 Venture CMOS
Absorption correction	Multi-scan (*SADABS*; Bruker, 2018 ▸)	Multi-scan (*SADABS*; Bruker, 2018 ▸)
*T*_min_, *T*_max_	0.470, 0.562	0.608, 0.745
No. of measured, independent and observed [*I* > 2σ(*I*)] reflections	44530, 3071, 2362	26314, 3038, 2868
*R*_int_	0.036	0.031
(sin θ/λ)_max_ (Å^−1^)	0.611	0.611

Refinement		
*R*[*F* ^2^ > 2σ(*F* ^2^)], *wR*(*F* ^2^), *S*	0.028, 0.054, 1.13	0.027, 0.071, 1.13
No. of reflections	3071	3038
No. of parameters	170	174
No. of restraints	1	5
H-atom treatment	H atoms treated by a mixture of independent and constrained refinement	H-atom parameters constrained
Δρ_max_, Δρ_min_ (e Å^−3^)	0.53, −0.47	0.46, −0.48
Absolute structure	–	Refined as an inversion twin
Absolute structure parameter	–	0.29 (5)

Computer programs: *APEX3* and *SAINT* (Bruker, 2018 ▸), *SHELXT2014* (Sheldrick, 2015*a* ▸), *SHELXL2018* (Sheldrick, 2015*b* ▸), *OLEX2* (Dolomanov *et al.*, 2009 ▸) and *publCIF* (Westrip, 2010 ▸).

## supplementary crystallographic information

### 2-(1H-Indol-3-yl)-N,N-dimethyl-N-propylazanium iodide (DMPT) Crystal data

**Table:**

C_15_H_23_N_2_^+^·I^−^	*F*(000) = 720
*M**_r_* = 358.25	*D*_x_ = 1.467 Mg m^−^^3^
Monoclinic, *P*2_1_/*c*	Mo *K*α radiation, λ = 0.71073 Å
*a* = 7.4471 (6) Å	Cell parameters from 9256 reflections
*b* = 9.9016 (9) Å	θ = 3.2–25.6°
*c* = 22.052 (2) Å	µ = 1.96 mm^−^^1^
β = 94.184 (3)°	*T* = 303 K
*V* = 1621.8 (2) Å^3^	Block, colourless
*Z* = 4	0.40 × 0.14 × 0.12 mm

### 2-(1H-Indol-3-yl)-N,N-dimethyl-N-propylazanium iodide (DMPT) Data collection

**Table:**

Bruker D8 Venture CMOS diffractometer	2362 reflections with *I* > 2σ(*I*)
φ and ω scans	*R*_int_ = 0.036
Absorption correction: multi-scan (SADABS; Bruker, 2018)	θ_max_ = 25.7°, θ_min_ = 3.2°
*T*_min_ = 0.470, *T*_max_ = 0.562	*h* = −9→8
44530 measured reflections	*k* = −12→12
3071 independent reflections	*l* = −26→26

### 2-(1H-Indol-3-yl)-N,N-dimethyl-N-propylazanium iodide (DMPT) Refinement

**Table:**

Refinement on *F*^2^	Hydrogen site location: mixed
Least-squares matrix: full	H atoms treated by a mixture of independent and constrained refinement
*R*[*F*^2^ > 2σ(*F*^2^)] = 0.028	*w* = 1/[σ^2^(*F*_o_^2^) + (0.0068*P*)^2^ + 2.2525*P*] where *P* = (*F*_o_^2^ + 2*F*_c_^2^)/3
*wR*(*F*^2^) = 0.054	(Δ/σ)_max_ < 0.001
*S* = 1.13	Δρ_max_ = 0.53 e Å^−^^3^
3071 reflections	Δρ_min_ = −0.47 e Å^−^^3^
170 parameters	Extinction correction: SHELXL2018 (Sheldrick, 2015b), Fc^*^=kFc[1+0.001xFc^2^λ^3^/sin(2θ)]^-1/4^
1 restraint	Extinction coefficient: 0.0054 (2)

### 2-(1H-Indol-3-yl)-N,N-dimethyl-N-propylazanium iodide (DMPT) Special details

**Table:**

Geometry. All esds (except the esd in the dihedral angle between two l.s. planes) are estimated using the full covariance matrix. The cell esds are taken into account individually in the estimation of esds in distances, angles and torsion angles; correlations between esds in cell parameters are only used when they are defined by crystal symmetry. An approximate (isotropic) treatment of cell esds is used for estimating esds involving l.s. planes.

### 2-(1H-Indol-3-yl)-N,N-dimethyl-N-propylazanium iodide (DMPT) Fractional atomic coordinates and isotropic or equivalent isotropic displacement parameters (Å^2^)

**Table:**

	*x*	*y*	*z*	*U*_iso_*/*U*_eq_	
I1	0.71085 (3)	0.75133 (2)	0.06875 (2)	0.04767 (10)	
N1	0.5983 (4)	0.7145 (3)	0.23013 (12)	0.0567 (8)	
H1	0.618 (5)	0.743 (4)	0.1943 (8)	0.068*	
N2	0.1783 (3)	0.7602 (3)	0.44696 (10)	0.0399 (6)	
C1	0.4437 (5)	0.7359 (4)	0.25813 (14)	0.0527 (8)	
H1A	0.351233	0.793238	0.243768	0.063*	
C2	0.7010 (4)	0.6220 (3)	0.26291 (13)	0.0428 (8)	
C3	0.8656 (5)	0.5669 (4)	0.25282 (17)	0.0576 (10)	
H3	0.926911	0.592411	0.219393	0.069*	
C4	0.9359 (5)	0.4738 (4)	0.2933 (2)	0.0648 (11)	
H4	1.045983	0.433742	0.286964	0.078*	
C5	0.8457 (5)	0.4376 (4)	0.34402 (18)	0.0614 (10)	
H5	0.897326	0.374036	0.371038	0.074*	
C6	0.6836 (4)	0.4930 (3)	0.35502 (14)	0.0461 (8)	
H6	0.625171	0.468037	0.389164	0.055*	
C7	0.6070 (4)	0.5878 (3)	0.31407 (12)	0.0359 (7)	
C8	0.4436 (4)	0.6624 (3)	0.30961 (13)	0.0411 (7)	
C9	0.2957 (4)	0.6588 (4)	0.35230 (15)	0.0499 (8)	
H9A	0.289023	0.569011	0.369594	0.060*	
H9B	0.181686	0.677469	0.329775	0.060*	
C10	0.3259 (4)	0.7604 (3)	0.40295 (13)	0.0409 (7)	
H10A	0.333745	0.849867	0.385399	0.049*	
H10B	0.440113	0.741329	0.425294	0.049*	
C11	−0.0058 (4)	0.7839 (3)	0.41756 (15)	0.0465 (8)	
H11A	−0.089932	0.788522	0.449056	0.056*	
H11B	−0.039201	0.706873	0.392006	0.056*	
C12	−0.0254 (5)	0.9091 (4)	0.37978 (16)	0.0557 (9)	
H12A	0.040947	0.898425	0.343839	0.067*	
H12B	0.026122	0.984880	0.402879	0.067*	
C13	−0.2223 (5)	0.9394 (4)	0.36047 (19)	0.0702 (11)	
H13A	−0.230110	1.022272	0.337814	0.105*	
H13B	−0.288996	0.947971	0.395921	0.105*	
H13C	−0.271725	0.867081	0.335514	0.105*	
C14	0.1764 (6)	0.6260 (4)	0.47924 (17)	0.0642 (11)	
H14A	0.144475	0.555988	0.450328	0.096*	
H14B	0.089724	0.628635	0.509391	0.096*	
H14C	0.293626	0.607988	0.498556	0.096*	
C15	0.2247 (5)	0.8653 (4)	0.49425 (15)	0.0533 (9)	
H15A	0.234238	0.951763	0.475042	0.080*	
H15B	0.337490	0.842968	0.515770	0.080*	
H15C	0.132085	0.868672	0.522304	0.080*	

### 2-(1H-Indol-3-yl)-N,N-dimethyl-N-propylazanium iodide (DMPT) Atomic displacement parameters (Å^2^)

**Table:**

	*U*^11^	*U*^22^	*U*^33^	*U*^12^	*U*^13^	*U*^23^
I1	0.05122 (14)	0.04717 (14)	0.04582 (14)	−0.00087 (11)	0.01182 (8)	−0.00478 (11)
N1	0.073 (2)	0.064 (2)	0.0345 (14)	0.0053 (16)	0.0152 (14)	0.0093 (14)
N2	0.0471 (14)	0.0354 (13)	0.0387 (13)	−0.0045 (13)	0.0129 (10)	−0.0017 (12)
C1	0.055 (2)	0.056 (2)	0.0470 (18)	0.0115 (18)	0.0010 (15)	0.0021 (18)
C2	0.0478 (18)	0.0453 (19)	0.0359 (17)	−0.0042 (15)	0.0081 (14)	−0.0101 (14)
C3	0.051 (2)	0.061 (2)	0.063 (2)	−0.0079 (19)	0.0175 (18)	−0.022 (2)
C4	0.039 (2)	0.064 (3)	0.090 (3)	0.0038 (18)	−0.002 (2)	−0.035 (2)
C5	0.062 (2)	0.051 (2)	0.066 (2)	0.0092 (19)	−0.025 (2)	−0.0099 (19)
C6	0.053 (2)	0.0460 (19)	0.0373 (17)	−0.0026 (16)	−0.0063 (14)	−0.0037 (15)
C7	0.0400 (16)	0.0390 (17)	0.0286 (15)	−0.0042 (14)	0.0010 (12)	−0.0052 (13)
C8	0.0430 (17)	0.0449 (18)	0.0357 (17)	0.0011 (14)	0.0057 (13)	−0.0051 (14)
C9	0.0435 (18)	0.052 (2)	0.055 (2)	−0.0039 (16)	0.0125 (15)	−0.0113 (16)
C10	0.0378 (15)	0.0432 (17)	0.0430 (16)	−0.0048 (15)	0.0106 (12)	−0.0006 (15)
C11	0.0450 (18)	0.049 (2)	0.0469 (18)	−0.0047 (14)	0.0119 (14)	−0.0026 (15)
C12	0.053 (2)	0.052 (2)	0.061 (2)	−0.0003 (17)	0.0026 (17)	0.0053 (18)
C13	0.061 (2)	0.074 (3)	0.074 (3)	0.000 (2)	−0.013 (2)	−0.004 (2)
C14	0.083 (3)	0.050 (2)	0.062 (2)	0.004 (2)	0.024 (2)	0.0159 (18)
C15	0.061 (2)	0.056 (2)	0.0422 (19)	−0.0003 (18)	0.0015 (16)	−0.0097 (17)

### 2-(1H-Indol-3-yl)-N,N-dimethyl-N-propylazanium iodide (DMPT) Geometric parameters (Å, º)

**Table:**

N1—H1	0.861 (10)	C9—H9A	0.9700
N1—C1	1.362 (4)	C9—H9B	0.9700
N1—C2	1.366 (4)	C9—C10	1.508 (4)
N2—C10	1.518 (3)	C10—H10A	0.9700
N2—C11	1.492 (4)	C10—H10B	0.9700
N2—C14	1.508 (4)	C11—H11A	0.9700
N2—C15	1.496 (4)	C11—H11B	0.9700
C1—H1A	0.9300	C11—C12	1.495 (4)
C1—C8	1.349 (4)	C12—H12A	0.9700
C2—C3	1.375 (5)	C12—H12B	0.9700
C2—C7	1.411 (4)	C12—C13	1.527 (5)
C3—H3	0.9300	C13—H13A	0.9600
C3—C4	1.361 (5)	C13—H13B	0.9600
C4—H4	0.9300	C13—H13C	0.9600
C4—C5	1.394 (6)	C14—H14A	0.9600
C5—H5	0.9300	C14—H14B	0.9600
C5—C6	1.364 (5)	C14—H14C	0.9600
C6—H6	0.9300	C15—H15A	0.9600
C6—C7	1.395 (4)	C15—H15B	0.9600
C7—C8	1.420 (4)	C15—H15C	0.9600
C8—C9	1.501 (4)		
			
C1—N1—H1	125 (3)	C10—C9—H9B	109.2
C1—N1—C2	108.9 (3)	N2—C10—H10A	108.9
C2—N1—H1	126 (3)	N2—C10—H10B	108.9
C11—N2—C10	114.0 (2)	C9—C10—N2	113.4 (2)
C11—N2—C14	107.7 (3)	C9—C10—H10A	108.9
C11—N2—C15	110.6 (2)	C9—C10—H10B	108.9
C14—N2—C10	109.6 (2)	H10A—C10—H10B	107.7
C15—N2—C10	107.7 (2)	N2—C11—H11A	108.5
C15—N2—C14	107.1 (3)	N2—C11—H11B	108.5
N1—C1—H1A	124.8	N2—C11—C12	115.0 (3)
C8—C1—N1	110.5 (3)	H11A—C11—H11B	107.5
C8—C1—H1A	124.8	C12—C11—H11A	108.5
N1—C2—C3	130.7 (3)	C12—C11—H11B	108.5
N1—C2—C7	107.0 (3)	C11—C12—H12A	109.3
C3—C2—C7	122.3 (3)	C11—C12—H12B	109.3
C2—C3—H3	121.1	C11—C12—C13	111.8 (3)
C4—C3—C2	117.9 (3)	H12A—C12—H12B	107.9
C4—C3—H3	121.1	C13—C12—H12A	109.3
C3—C4—H4	119.5	C13—C12—H12B	109.3
C3—C4—C5	121.0 (3)	C12—C13—H13A	109.5
C5—C4—H4	119.5	C12—C13—H13B	109.5
C4—C5—H5	119.2	C12—C13—H13C	109.5
C6—C5—C4	121.6 (3)	H13A—C13—H13B	109.5
C6—C5—H5	119.2	H13A—C13—H13C	109.5
C5—C6—H6	120.6	H13B—C13—H13C	109.5
C5—C6—C7	118.7 (3)	N2—C14—H14A	109.5
C7—C6—H6	120.6	N2—C14—H14B	109.5
C2—C7—C8	107.0 (3)	N2—C14—H14C	109.5
C6—C7—C2	118.4 (3)	H14A—C14—H14B	109.5
C6—C7—C8	134.6 (3)	H14A—C14—H14C	109.5
C1—C8—C7	106.6 (3)	H14B—C14—H14C	109.5
C1—C8—C9	125.9 (3)	N2—C15—H15A	109.5
C7—C8—C9	127.5 (3)	N2—C15—H15B	109.5
C8—C9—H9A	109.2	N2—C15—H15C	109.5
C8—C9—H9B	109.2	H15A—C15—H15B	109.5
C8—C9—C10	111.9 (3)	H15A—C15—H15C	109.5
H9A—C9—H9B	107.9	H15B—C15—H15C	109.5
C10—C9—H9A	109.2		
			
N1—C1—C8—C7	−1.0 (4)	C3—C4—C5—C6	0.4 (5)
N1—C1—C8—C9	−179.5 (3)	C4—C5—C6—C7	0.2 (5)
N1—C2—C3—C4	−179.0 (3)	C5—C6—C7—C2	0.1 (4)
N1—C2—C7—C6	179.4 (3)	C5—C6—C7—C8	178.2 (3)
N1—C2—C7—C8	0.9 (3)	C6—C7—C8—C1	−178.1 (3)
N2—C11—C12—C13	−170.0 (3)	C6—C7—C8—C9	0.3 (6)
C1—N1—C2—C3	179.1 (3)	C7—C2—C3—C4	1.7 (5)
C1—N1—C2—C7	−1.5 (4)	C7—C8—C9—C10	89.1 (4)
C1—C8—C9—C10	−92.7 (4)	C8—C9—C10—N2	179.7 (3)
C2—N1—C1—C8	1.6 (4)	C10—N2—C11—C12	−54.3 (4)
C2—C3—C4—C5	−1.3 (5)	C11—N2—C10—C9	−56.6 (4)
C2—C7—C8—C1	0.1 (3)	C14—N2—C10—C9	64.1 (3)
C2—C7—C8—C9	178.6 (3)	C14—N2—C11—C12	−176.1 (3)
C3—C2—C7—C6	−1.1 (5)	C15—N2—C10—C9	−179.7 (3)
C3—C2—C7—C8	−179.7 (3)	C15—N2—C11—C12	67.2 (3)

### 2-(1H-Indol-3-yl)-N,N-dimethyl-N-propylazanium iodide (DMPT) Hydrogen-bond geometry (Å, º)

**Table:**

*D*—H···*A*	*D*—H	H···*A*	*D*···*A*	*D*—H···*A*
N1—H1···I1	0.86 (1)	2.91 (2)	3.733 (3)	162 (3)

### 2-(1H-Indol-3-yl)-N,N-dimethyl-N-(prop-2-en-1-yl)azanium iodide (DMALT) Crystal data

**Table:**

0.5C_15_H_21_N_2_^+^·0.5I^−^	*F*(000) = 356
*M**_r_* = 178.12	*D*_x_ = 1.480 Mg m^−^^3^
Monoclinic, *P*2_1_	Mo *K*α radiation, λ = 0.71073 Å
*a* = 7.3471 (8) Å	Cell parameters from 9625 reflections
*b* = 9.9672 (9) Å	θ = 3.2–25.7°
*c* = 10.9499 (11) Å	µ = 1.99 mm^−^^1^
β = 94.671 (3)°	*T* = 303 K
*V* = 799.20 (14) Å^3^	Block, colourless
*Z* = 4	0.39 × 0.22 × 0.15 mm

### 2-(1H-Indol-3-yl)-N,N-dimethyl-N-(prop-2-en-1-yl)azanium iodide (DMALT) Data collection

**Table:**

Bruker D8 Venture CMOS diffractometer	2868 reflections with *I* > 2σ(*I*)
φ and ω scans	*R*_int_ = 0.031
Absorption correction: multi-scan (SADABS; Bruker, 2018)	θ_max_ = 25.7°, θ_min_ = 3.2°
*T*_min_ = 0.608, *T*_max_ = 0.745	*h* = −8→8
26314 measured reflections	*k* = −12→12
3038 independent reflections	*l* = −13→13

### 2-(1H-Indol-3-yl)-N,N-dimethyl-N-(prop-2-en-1-yl)azanium iodide (DMALT) Refinement

**Table:**

Refinement on *F*^2^	H-atom parameters constrained
Least-squares matrix: full	*w* = 1/[σ^2^(*F*_o_^2^) + (0.0345*P*)^2^ + 0.614*P*] where *P* = (*F*_o_^2^ + 2*F*_c_^2^)/3
*R*[*F*^2^ > 2σ(*F*^2^)] = 0.027	(Δ/σ)_max_ = 0.001
*wR*(*F*^2^) = 0.071	Δρ_max_ = 0.46 e Å^−^^3^
*S* = 1.13	Δρ_min_ = −0.48 e Å^−^^3^
3038 reflections	Extinction correction: SHELXL2018 (Sheldrick, 2015b), Fc^*^=kFc[1+0.001xFc^2^λ^3^/sin(2θ)]^-1/4^
174 parameters	Extinction coefficient: 0.056 (3)
5 restraints	Absolute structure: Refined as an inversion twin
Hydrogen site location: inferred from neighbouring sites	Absolute structure parameter: 0.29 (5)

### 2-(1H-Indol-3-yl)-N,N-dimethyl-N-(prop-2-en-1-yl)azanium iodide (DMALT) Special details

**Table:**

Geometry. All esds (except the esd in the dihedral angle between two l.s. planes) are estimated using the full covariance matrix. The cell esds are taken into account individually in the estimation of esds in distances, angles and torsion angles; correlations between esds in cell parameters are only used when they are defined by crystal symmetry. An approximate (isotropic) treatment of cell esds is used for estimating esds involving l.s. planes.
Refinement. Refined as a 2-component inversion twin.

### 2-(1H-Indol-3-yl)-N,N-dimethyl-N-(prop-2-en-1-yl)azanium iodide (DMALT) Fractional atomic coordinates and isotropic or equivalent isotropic displacement parameters (Å^2^)

**Table:**

	*x*	*y*	*z*	*U*_iso_*/*U*_eq_	Occ. (<1)
I1	0.30017 (4)	0.61487 (8)	0.86560 (3)	0.05386 (18)	
N1	0.3947 (9)	0.5802 (8)	0.5378 (5)	0.080 (3)	
H1	0.366598	0.619421	0.603678	0.096*	
N2	0.8350 (6)	0.6202 (10)	0.1131 (4)	0.0491 (11)	
C1	0.5456 (9)	0.6076 (16)	0.4777 (6)	0.077 (2)	
H1A	0.633090	0.672620	0.499495	0.093*	
C2	0.2924 (9)	0.4792 (7)	0.4766 (5)	0.0498 (14)	
C3	0.1281 (10)	0.4191 (8)	0.5025 (7)	0.0669 (19)	
H3	0.068939	0.443978	0.570894	0.080*	
C4	0.0577 (10)	0.3229 (9)	0.4240 (9)	0.076 (2)	
H4	−0.051395	0.281303	0.439444	0.091*	
C5	0.1458 (11)	0.2850 (8)	0.3203 (8)	0.074 (2)	
H5	0.094346	0.219554	0.267657	0.089*	
C6	0.3082 (10)	0.3444 (7)	0.2962 (6)	0.0589 (16)	
H6	0.366647	0.318328	0.227823	0.071*	
C7	0.3847 (8)	0.4426 (6)	0.3731 (5)	0.0445 (12)	
C8	0.5458 (9)	0.5203 (7)	0.3770 (6)	0.0576 (16)	
C9	0.6952 (11)	0.5117 (9)	0.2917 (8)	0.074 (2)	
H9A	0.812499	0.516278	0.339083	0.089*	
H9B	0.687847	0.425695	0.250073	0.089*	
C10	0.6846 (8)	0.6175 (13)	0.2017 (5)	0.0589 (15)	
H10A	0.685969	0.702654	0.244613	0.071*	
H10B	0.567847	0.610421	0.153994	0.071*	
C13	1.0232 (7)	0.6147 (14)	0.1727 (5)	0.0670 (14)	
H13A	1.106539	0.583631	0.114432	0.080*	0.30 (4)
H13B	1.028342	0.552140	0.240738	0.080*	0.30 (4)
H13C	1.108633	0.616136	0.109644	0.080*	0.70 (4)
H13D	1.039323	0.530235	0.216237	0.080*	0.70 (4)
C12	0.8059 (14)	0.7411 (10)	0.0302 (8)	0.055 (2)	
H12A	0.822133	0.821611	0.078022	0.083*	
H12B	0.684335	0.739046	−0.009237	0.083*	
H12C	0.892702	0.739452	−0.030715	0.083*	
C14	1.080 (7)	0.755 (3)	0.219 (4)	0.084 (5)	0.30 (4)
H14	0.995335	0.818264	0.189308	0.101*	0.30 (4)
C15	1.216 (7)	0.813 (8)	0.290 (4)	0.090 (5)	0.30 (4)
H15A	1.311433	0.761751	0.326014	0.108*	0.30 (4)
H15B	1.215032	0.905645	0.302622	0.108*	0.30 (4)
C14A	1.070 (2)	0.7277 (18)	0.2611 (18)	0.084 (5)	0.70 (4)
H14A	1.001641	0.742461	0.327829	0.101*	0.70 (4)
C15A	1.211 (3)	0.806 (3)	0.241 (2)	0.090 (5)	0.70 (4)
H15C	1.277982	0.789671	0.173504	0.108*	0.70 (4)
H15D	1.243546	0.876433	0.293577	0.108*	0.70 (4)
C11	0.811 (2)	0.5005 (13)	0.0358 (14)	0.094 (5)	
H11A	0.689549	0.499355	−0.003742	0.140*	
H11B	0.830123	0.421667	0.085540	0.140*	
H11C	0.897905	0.502045	−0.025164	0.140*	

### 2-(1H-Indol-3-yl)-N,N-dimethyl-N-(prop-2-en-1-yl)azanium iodide (DMALT) Atomic displacement parameters (Å^2^)

**Table:**

	*U*^11^	*U*^22^	*U*^33^	*U*^12^	*U*^13^	*U*^23^
I1	0.0558 (2)	0.0573 (2)	0.0503 (2)	−0.0009 (3)	0.01514 (13)	0.0030 (3)
N1	0.074 (4)	0.120 (8)	0.048 (3)	−0.014 (4)	0.020 (3)	−0.026 (3)
N2	0.053 (2)	0.046 (2)	0.050 (2)	0.020 (4)	0.0160 (17)	0.011 (4)
C1	0.064 (4)	0.100 (6)	0.068 (4)	−0.029 (7)	0.012 (3)	−0.033 (7)
C2	0.053 (3)	0.057 (4)	0.042 (3)	0.000 (3)	0.014 (3)	0.003 (3)
C3	0.058 (4)	0.074 (5)	0.072 (5)	0.005 (4)	0.023 (3)	0.018 (4)
C4	0.045 (4)	0.078 (5)	0.101 (6)	−0.009 (3)	−0.004 (4)	0.031 (5)
C5	0.071 (5)	0.065 (4)	0.080 (5)	−0.015 (4)	−0.027 (4)	0.014 (4)
C6	0.071 (4)	0.056 (4)	0.048 (4)	0.008 (3)	−0.007 (3)	0.000 (3)
C7	0.047 (3)	0.050 (3)	0.036 (3)	0.002 (2)	0.002 (2)	0.000 (2)
C8	0.052 (4)	0.067 (4)	0.056 (4)	−0.002 (3)	0.018 (3)	0.000 (3)
C9	0.065 (4)	0.077 (5)	0.086 (5)	0.012 (4)	0.034 (4)	0.017 (4)
C10	0.055 (3)	0.065 (3)	0.059 (3)	0.029 (6)	0.019 (2)	0.004 (6)
C13	0.053 (3)	0.089 (4)	0.062 (3)	0.008 (8)	0.019 (2)	0.010 (8)
C12	0.066 (6)	0.057 (5)	0.042 (4)	0.000 (4)	−0.001 (4)	0.017 (4)
C14	0.072 (6)	0.144 (11)	0.040 (9)	−0.009 (7)	0.025 (7)	−0.014 (9)
C15	0.072 (6)	0.115 (9)	0.081 (14)	0.009 (6)	0.001 (11)	0.006 (14)
C14A	0.072 (6)	0.144 (11)	0.040 (9)	−0.009 (7)	0.025 (7)	−0.014 (9)
C15A	0.072 (6)	0.115 (9)	0.081 (14)	0.009 (6)	0.001 (11)	0.006 (14)
C11	0.110 (10)	0.058 (6)	0.121 (11)	−0.015 (6)	0.062 (8)	−0.027 (6)

### 2-(1H-Indol-3-yl)-N,N-dimethyl-N-(prop-2-en-1-yl)azanium iodide (DMALT) Geometric parameters (Å, º)

**Table:**

N1—H1	0.8600	C9—C10	1.441 (12)
N1—C1	1.363 (9)	C10—H10A	0.9700
N1—C2	1.396 (10)	C10—H10B	0.9700
N2—C10	1.529 (6)	C13—H13A	0.9700
N2—C13	1.482 (7)	C13—H13B	0.9700
N2—C12	1.513 (13)	C13—H13C	0.9700
N2—C11	1.465 (16)	C13—H13D	0.9700
C1—H1A	0.9300	C13—C14	1.530 (14)
C1—C8	1.405 (11)	C13—C14A	1.506 (12)
C2—C3	1.397 (10)	C12—H12A	0.9600
C2—C7	1.415 (8)	C12—H12B	0.9600
C3—H3	0.9300	C12—H12C	0.9600
C3—C4	1.361 (13)	C14—H14	0.9300
C4—H4	0.9300	C14—C15	1.349 (14)
C4—C5	1.404 (13)	C15—H15A	0.9300
C5—H5	0.9300	C15—H15B	0.9300
C5—C6	1.377 (11)	C14A—H14A	0.9300
C6—H6	0.9300	C14A—C15A	1.334 (12)
C6—C7	1.380 (9)	C15A—H15C	0.9300
C7—C8	1.412 (9)	C15A—H15D	0.9300
C8—C9	1.501 (9)	C11—H11A	0.9600
C9—H9A	0.9700	C11—H11B	0.9600
C9—H9B	0.9700	C11—H11C	0.9600
			
C1—N1—H1	125.1	C9—C10—N2	116.4 (6)
C1—N1—C2	109.9 (6)	C9—C10—H10A	108.2
C2—N1—H1	125.1	C9—C10—H10B	108.2
C13—N2—C10	114.6 (4)	H10A—C10—H10B	107.3
C13—N2—C12	112.0 (8)	N2—C13—H13A	109.8
C12—N2—C10	108.7 (7)	N2—C13—H13B	109.8
C11—N2—C10	107.1 (9)	N2—C13—H13C	108.7
C11—N2—C13	106.7 (9)	N2—C13—H13D	108.7
C11—N2—C12	107.3 (6)	N2—C13—C14	109 (2)
N1—C1—H1A	126.0	N2—C13—C14A	114.2 (11)
N1—C1—C8	107.9 (8)	H13A—C13—H13B	108.2
C8—C1—H1A	126.0	H13C—C13—H13D	107.6
N1—C2—C3	130.7 (6)	C14—C13—H13A	109.8
N1—C2—C7	107.2 (5)	C14—C13—H13B	109.8
C3—C2—C7	122.1 (6)	C14A—C13—H13C	108.7
C2—C3—H3	121.2	C14A—C13—H13D	108.7
C4—C3—C2	117.6 (7)	N2—C12—H12A	109.5
C4—C3—H3	121.2	N2—C12—H12B	109.5
C3—C4—H4	119.2	N2—C12—H12C	109.5
C3—C4—C5	121.6 (7)	H12A—C12—H12B	109.5
C5—C4—H4	119.2	H12A—C12—H12C	109.5
C4—C5—H5	119.9	H12B—C12—H12C	109.5
C6—C5—C4	120.2 (7)	C13—C14—H14	110.4
C6—C5—H5	119.9	C15—C14—C13	139 (5)
C5—C6—H6	119.8	C15—C14—H14	110.4
C5—C6—C7	120.3 (7)	C14—C15—H15A	120.0
C7—C6—H6	119.8	C14—C15—H15B	120.0
C6—C7—C2	118.2 (6)	H15A—C15—H15B	120.0
C6—C7—C8	134.9 (6)	C13—C14A—H14A	121.1
C8—C7—C2	106.9 (5)	C15A—C14A—C13	118 (2)
C1—C8—C7	108.0 (6)	C15A—C14A—H14A	121.1
C1—C8—C9	124.9 (7)	C14A—C15A—H15C	120.0
C7—C8—C9	127.1 (7)	C14A—C15A—H15D	120.0
C8—C9—H9A	109.1	H15C—C15A—H15D	120.0
C8—C9—H9B	109.1	N2—C11—H11A	109.5
H9A—C9—H9B	107.8	N2—C11—H11B	109.5
C10—C9—C8	112.5 (6)	N2—C11—H11C	109.5
C10—C9—H9A	109.1	H11A—C11—H11B	109.5
C10—C9—H9B	109.1	H11A—C11—H11C	109.5
N2—C10—H10A	108.2	H11B—C11—H11C	109.5
N2—C10—H10B	108.2		
			
N1—C1—C8—C7	3.0 (12)	C4—C5—C6—C7	−0.6 (11)
N1—C1—C8—C9	−175.9 (8)	C5—C6—C7—C2	0.4 (9)
N1—C2—C3—C4	178.9 (7)	C5—C6—C7—C8	178.6 (7)
N1—C2—C7—C6	−179.2 (6)	C6—C7—C8—C1	178.5 (9)
N1—C2—C7—C8	2.1 (7)	C6—C7—C8—C9	−2.6 (13)
N2—C13—C14—C15	170 (6)	C7—C2—C3—C4	−0.1 (11)
N2—C13—C14A—C15A	−121.9 (19)	C7—C8—C9—C10	101.8 (10)
C1—N1—C2—C3	−179.4 (9)	C8—C9—C10—N2	178.0 (7)
C1—N1—C2—C7	−0.2 (10)	C10—N2—C13—C14	−80.6 (19)
C1—C8—C9—C10	−79.5 (12)	C10—N2—C13—C14A	−58.9 (14)
C2—N1—C1—C8	−1.8 (12)	C13—N2—C10—C9	−51.2 (12)
C2—C3—C4—C5	−0.2 (11)	C12—N2—C10—C9	−177.3 (8)
C2—C7—C8—C1	−3.1 (9)	C12—N2—C13—C14	43.9 (18)
C2—C7—C8—C9	175.8 (7)	C12—N2—C13—C14A	65.5 (12)
C3—C2—C7—C6	0.0 (9)	C11—N2—C10—C9	67.0 (11)
C3—C2—C7—C8	−178.7 (7)	C11—N2—C13—C14	161.1 (18)
C3—C4—C5—C6	0.6 (12)	C11—N2—C13—C14A	−177.3 (12)

### 2-(1H-Indol-3-yl)-N,N-dimethyl-N-(prop-2-en-1-yl)azanium iodide (DMALT) Hydrogen-bond geometry (Å, º)

**Table:**

*D*—H···*A*	*D*—H	H···*A*	*D*···*A*	*D*—H···*A*
N1—H1···I1	0.86	2.95	3.727 (6)	152
